# High-Precision Fast-Spiking Basket Cell Discharges during Complex Events in the Human Neocortex

**DOI:** 10.1523/ENEURO.0260-17.2017

**Published:** 2017-10-13

**Authors:** Viktor Szegedi, Gábor Molnár, Melinda Paizs, Eszter Csakvari, Pál Barzó, Gábor Tamás, Karri Lamsa

**Affiliations:** 1 MTA-NAP Research Group for Inhibitory Interneurons and Plasticity, Department of Physiology, Anatomy and Neuroscience, University of Szeged, Szeged 6726, Hungary; 2 MTA-SZTE Research Group for Cortical Microcircuits, Department of Physiology, Anatomy and Neuroscience, University of Szeged, Szeged 6726, Hungary; 3 Department of Neurosurgery, University of Szeged, Szeged 6725, Hungary

**Keywords:** gamma oscillation, human cognition, neuronal ensemble

## Abstract

In the human neocortex, solitary action potentials in some layer 2–3 pyramidal cells (PCs) trigger brief episodes of network activity known as complex events through strong excitatory synapses that specifically innervate GABAergic interneurons. Yet, how these “master PCs” configure the local network activity is not well understood. We report that single spikes in the PCs, studied here in synaptically connected cell pairs in frontal or temporal neocortical areas of both males and females, elicit firing of fast-spiking basket cells (FSBCs) with a short delay (on average 2.7 ms). The FSBC discharge is triggered by 13 mV (on average) monosynaptic EPSPs, and the action potential is time locked to the master PC spike with high temporal precision, showing little jitter in delay. In the complex events, the FSBC discharge occurs in the beginning of the activity episode, forming the first wave of the complex event activity. Firing of FSBCs generates GABAergic IPSCs with fast kinetics in layer 2–3 PCs, and similar IPSCs regularly occur time locked to master PC spikes in the beginning of the complex events with high probability and short (median 4.1 ms) delay with little jitter. In comparison, discharge of nonfast spiking interneurons (non-FSINs) investigated here appears inconsistently in the complex events and shows low probability. Thus, firing of layer 2–3 FSBCs with high temporal fidelity characterizes early phase of the complex events in the human neocortex.

## Significance Statement

In the human neocortex solitary action potentials of some pyramidal cells (PCs) trigger network activity episodes known as complex events. These “master PCs” with remarkably strong synapses occur widely in the human neocortical layers 2 and 3, but are not found in rodent neocortex and little is known about the network activity they evoke. We report that the master PCs configure neocortical network activity in a precise manner by activating specialized inhibitory interneurons, fast-spiking basket cells (FSBCs), in the beginning of the complex events with an accurate temporal pattern. Temporally patterned high-precision firing of FSBCs is a hallmark of many physiologic processes in the neocortex, and our results show that solitary PC spikes can initiate such activity in humans.

## Introduction

Information in the neocortex is encoded by the temporally organized discharge of neuronal ensembles, and this requires timed activation of specialized GABAergic interneurons (Ainsworth et al., 2012; Buzsaki and Watson, 2012). Human neocortical microcircuits show a low threshold for generation of small-scale neuronal population activity, because strong multivesicular excitatory synapses connect some layer 2–3 pyramidal cells (PCs) to GABAergic interneurons with very large suprathreshold EPSPs (VLEs). Consequently, a solitary spike in the “master PC” triggers firing in the local interneurons, initiating a tens-of-milliseconds-long population burst known as a complex event (Molnár et al., 2008; Brecht, 2012; Molnar et al., 2016; Szegedi et al., 2016). Although the complex events occur in various neocortical areas in humans, similar solitary PC spike-evoked network activity episodes have not been reported in the rodent neocortex (Molnár et al., 2008; Komlosi et al., 2012; Doron and Brecht, 2015; Molnar et al., 2016; Szegedi et al., 2016; Lourenço and Bacci, 2017). A specific role of the complex events in the human neocortical microcircuits is unknown, but it has been proposed that master PCs may have evolved in the evolutionary process to support generation of neuronal ensembles in higher-order cerebral functions (Komlosi et al., 2012; Lourenço and Bacci, 2017). If this hypothesis is correct, one would also expect the complex events to exhibit temporal patterns in discharge of the neurons, as temporally structured firing characterizes neuronal ensembles (Isaacson and Scanziani, 2011; Brecht, 2012; Buzsaki and Watson, 2012). Hence, we investigate here whether the master PC-evoked complex events show temporally organized discharge of a specific GABAergic interneuron type, the fast-spiking basket cell (FSBC). The FSBCs have a well-established role in generation of coordinated cortical high-frequency network activities involved in cognitive processes and they are key players in the neuronal ensemble activity (Buzsaki and Watson, 2012; Lewis et al., 2012). The experiments are conducted in slices from neocortical tissue resected in surgeries for the operation of subcortical or deep cortical targets. First, we investigate the FSBC firing delay and the action potential temporal precision elicited by solitary master PC spikes. Second, we examine GABAergic output from the FSBCs and some nonfast spiking interneurons (non-FSINs) during master PC-evoked complex events using dual recordings from PCs. The results show that master PC spikes evoke high-precision firing of the FSBCs, and that the FSBCs are activated in the first wave of GABAergic activity in the complex events. We conclude that the short-delay discharge of FSBCs with high temporal precision is a regular feature of master PC-evoked complex events in the human neocortex.

## Materials and Methods

### Ethics statement

All procedures were performed according to the Declaration of Helsinki with the approval of the University of Szeged Ethical Committee and Regional Human Investigation Review Board (ref. 75/2014). None of the experiments were reported before with a minor exception that in five of the fifteen cells reporting monosynaptic IPSCs one data parameter (IPSC amplitude) has been included in a previous manuscript (Szegedi et al., 2016). However, the other data parameters of these cells (rise slope, normalized slope) have not been reported before.

### Brain slices

Human neocortical slices were derived from material that had to be removed to gain access for the surgical treatment of deep-brain targets (tumor, cyst, aneurysm, or catheter implant) from the left and right frontal, temporal, and parietal regions, with written informed consent of the patients before surgery. In some cases, tissue from neocortical operations was used when it was nonpathologic. In these latter cases, small pieces of nonpathologic tissue had to be removed in the surgery to get access to pathologic targets in the folded neocortex. The patients were 10–60 years of age, including 21 and 18 samples from males and females, respectively. The tissue obtained from underage patients was provided with agreement from a parent or guardian. Details including the patient gender, age, the resected neocortical area and the pathologic target diagnosis are reported for all tissue samples used in this study in Table 1. Anesthesia was induced with intravenous midazolam and fentanyl (0.03 mg/kg and 1–2 µ/kg, respectively). A bolus dose of propofol (1–2 mg/kg) was administered intravenously. The patients received 0.5 mg/kg rocuronium to facilitate endotracheal intubation. The trachea was intubated and the patient was ventilated with O_2_/N_2_O mixture at a ratio of 1:2. Anesthesia was maintained with sevoflurane at care volume of 1.2–1.5. Following surgical removal, the resected tissue blocks were immediately immersed into a glass container filled with ice-cold solution in the operating theater. The solution contained 130 mM NaCl, 3.5 mM KCl, 1 mM NaH_2_PO_4_, 24 mM NaHCO_3_, 1 mM CaCl_2_, 3 mM MgSO_4_, and 10 mM D(+)-glucose, and was saturated with 95% O_2_/5% CO_2_. The container was placed on ice in a thermally isolated transportation box where the liquid was continuously gassed with 95% O_2_/5% CO_2_. Then, the tissue was transported from the operating theater to the electrophysiology lab (door-to-door in maximum 20 min), where slices of 350-μm thickness were immediately prepared from the block with a vibrating blade microtome (Microm HM 650 V). The slices were incubated at room temperature (22–24°C) for 1 h, when the slicing solution was gradually replaced by a pump (6 ml/min) with the solution used for storage (180 ml, content identical to a solution used in electrophysiology experiments). The storage solution was identical to the slicing solution, except containing 3 mM CaCl_2_ and 1.5 mM MgSO_4_.

**Table 1.** Details showing the patient gender, age and the resected cortical area of the tissue samples used in the experiments of this study

Cell ID	Figure showing the data	Gender	Age (years)	Hemisphere	Neocortical area (material removed to gain access to surgical treatment of pathological targets)	Experiment code	Diagnosed pathology
FSBC 1	Fig. 1*A*,*B*	Male	49	Left	Temporal	0501043j	Cortical and subcortical neoplasm
FSBC 2	Fig. 1*C*	Female	42	Left	Frontal	K1901171	Subcortical neoplasm
FSBC 3	Fig. 1*D*	Male	43	Left	Temporal	1403062	Subcortical neoplasm
FSBC 4	Fig. 1*E*	Male	29	Right	Frontal	0609121s	Cortical and subcortical neoplasm
FSBC 5	Fig. 1*F*	Male	54	Right	Temporal	0705173s	Cortical and subcortical neoplasm
non-FSIN 1	Fig. 2*A*	Male	58	Right	Temporal	0512022	Subcortical neoplasm
non-FSIN 2	Fig. 2*B*	Female	68	Right	Temporoparietal	k0205171	Cortical and subcortical metaplasia
non-FSIN 3	Fig. 2*C*	Female	64	Right	Frontal	0601171	Subcortical neoplasm
Cell pair 1	Fig. 3*A*	Male	40	Left	Temporal	1405151	Subcortical neoplasm
Cell pair 2	Fig. 3*B*	Male	58	Left	Temporal	1509122	Subcortical neoplasm
Cell pair 3	Fig. 3*B*	Male	36	Left	Temporal	1311131	Subcortical neoplasm
Cell pair 4	Fig. 3*B*	Male	17	Left	Parieto-occipital	1110271	Cortical and subcortical neoplasm
Cell pair 5	Fig. 3*B*	Male	48	Right	Frontal	1401233	Subcortical aneurysm
Cell pair 6	Fig. 3*B*	Male	40	Left	Temporal	1405152	Subcortical neoplasm
Cell pair 7	Fig. 3*B*	Male	49	Right	Frontal	1310092	Meningioma
Cell pair 8	Fig. 3*B*	Male	36	Right	Temporal	1112082	Subcortical neoplasm
Cell pair 9	Fig. 3*B*	Male	16	Right	Parieto-occipital	1402181	Subcortical neoplasm
Cell pair 10	Fig. 3*C*	Female	33	Right	Temporal	1510301	Cortical and subcortical neoplasm
pv+ BC 1	Fig. 4*A*	Male	55	Right	Frontal	k0409152	Cortical and subcortical neoplasm
pv+ BC 2	Fig. 4*A*	Female	10	Left	Frontal	k2506151	Subcortical neoplasm
pv+ BC 3	Fig. 4*A*	Female	10	Left	Frontal	k2506155	Subcortical neoplasm
pv+ BC 4	Fig. 4*A*	Female	30	Left	Parieto-occipital	k2306151	Shunt for hydrocephalus
pv+ BC 5	Fig. 4*A*	Female	40	Right	Frontal	k2309153	Subcortical neoplasm
pv+ BC 6	Fig. 4*A*	Female	28	Right	Parieto-occipital	k2804151	Subcortical neoplasm
uFS 1	Fig. 4*A*	Female	67	Right	Frontal	100306c11	Epidural hemorrhage
uFS 2	Fig. 4*A*	Male	55	Right	Frontal	040915c11	Cortical and subcortical neoplasm
uFS 3	Fig. 4*A*	Male	47	Right	Frontal	021005c3	Cortical and subcortical metaplasia
uFS 4	Fig. 4*A*	Female	59	Right	Frontal	K2510161	Shunt for hydrocephalus
non-FS 1	Fig. 4*A*	Female	33	Right	Temporal	301015c1	Cortical and subcortical neoplasm
non-FS 2	Fig. 4*A*	Male	19	Right	Parieto-occipital	151015c3	Shunt for hydrocephalus
non-FS 3	Fig. 4*A*	Female	33	Left	Parieto-occipital	051115c7	Meningioma
non-FS 4	Fig. 4*A*	Female	37	Right	Temporal	050416t6	Subcortical neoplasm
non-FS 5	Fig. 4*A*	Male	47	Right	Frontal	021015c12	Subcortical neoplasm
Cell pair 1	Fig. 4*C*	Female	55	Left	Frontal	k1208152	Shunt for hydrocephalus
Cell pair 2	Fig. 4*C*	Male	58	Left	Temporal	k0109151	Subcortical neoplasm
Cell pair 3	Fig. 4*C*	Female	50	Right	Frontal	k2511161	Subcortical neoplasm
Cell pair 4	Fig. 4*C*	Female	33	Left	Parieto-occipital	k0511151	Meningioma
Cell pair 5	Fig. 4*D*	Female	55	Left	Temporal	k1208151	Subcortical neoplasm
Cell pair 6	Fig. 4*D*	Male	60	Left	Temporal	k2806161	Subcortical neoplasm

Cell ID refers to the cell pair code used in the text and in the figures. Second column identifies the figure in which the specific experiment data are illustrated. Experiment code refers to original identification number of the cell pair in the authors’ files, and last column shows patient pathology diagnosed for the surgery.

### Electrophysiology

**Figure 1.**
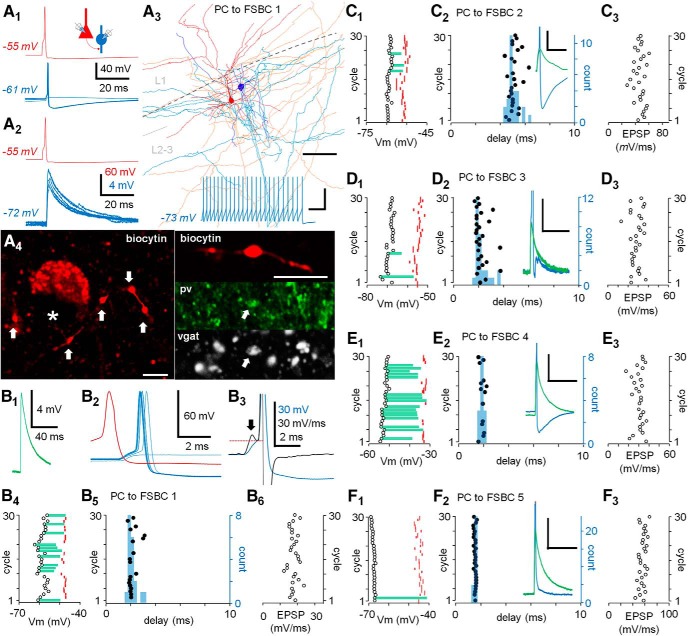
Very large monosynaptic EPSP from single PC triggers short-delay high-precision discharge of FSBCs. ***A***, ***B***, Solitary master PC (red) spikes trigger firing in a postsynaptic FSBC (blue) with a short delay and high temporal fidelity through very large monosynaptic EPSP (VLEs). ***A1***, Single PC spikes (elicited with 2- to 3-ms suprathreshold depolarizing pulses) trigger discharge in FSBC 1 (Vm = –61 mV) with occasional failures (six consecutive responses superimposed). Schematic summarizes experimental design. ***A2***, FSBC 1 hyperpolarization precludes the action potential revealing monosynaptic VLE (blue, six consecutive responses superimposed). ***A3***, Illustration of the PC (soma and dendrites red, axon orange) to FSBC 1 (blue, axon light blue) pair with VLEs. L1 and L2-3: layer 1 and 2–3, respectively. Scale bar: 100 μm. Inset below shows the FSBC 1 firing response without apparent firing accommodation (600-ms depolarizing pulse). Scale bars: 60 mV, 100 ms. ***A4***, Biocytin-filled postsynaptic FSBC (FSBC 2) axon in L2-3. Left, Axon boutons (indicated by arrows) are arranged around an unlabelled L2-3 cell soma (asterisk, endofluorescence in nucleus). Right, Biocytin (Cy3)-filled bouton with positive immunoreaction (arrow) for pv (Alexa Fluor 488) and vgat (Cy5) in the same cell. Scale bar: 5 μm. ***B1***, Average of the VLEs that failed to fire (six) in the FSBC 1. ***B2***, Consecutive VLEs each eliciting single action potential in the FSBC 1 (blue, six events including a VLE that failed to fire) by solitary PC spikes (10-s interval, one sample shown in red). ***B3***, Derivative (black line) of a VLE with spike (blue line). Arrow indicates the VLE maximum rise slope, and the following hump in the derivative corresponds to the action potential onset. The onset membrane potential (Vm) is indicated by horizontal dotted red line. ***B4***, Firing of the FSBC 1 by VLEs for 30 consecutive PC spikes (10-s interval). Open circles show the FSBC 1 membrane potential (Vm). Red marks show Vm for the onset of the triggered action potentials. Green bars illustrate the amplitude of the VLEs that failed to fire. ***B5***, Timing of the FSBC 1 firing (black dots) in the 30 consecutive cycles. Blue histogram summarizes the evoked spike delay distribution (count, bin 0.25 ms). ***B6***, The VLE maximum rise slope plotted for the 30 consecutive responses (as in ***B2***, ***B4***). ***C–F***, High temporal fidelity characterizes spike transmission in PC-FSBC pairs connected with VLEs. Experiments as in ***A***, ***B*** in four PC-FSBC pairs (FSBC 2–5) showing VLEs. ***C1***, FSBC 2 membrane potential (Vm) in 30 consecutive cycles of PC spike. Red marks, membrane potential (Vm) for the onset of the postsynaptic action potentials. Green bars show amplitude of the VLEs that failed to fire. ***C2***, Timing of the FSBC 2 firing (black dots) in the 30 cycles. Histogram (blue, bin 0.25 ms) summarizes the spike delay distribution. Inset, Two sample traces in the experiment showing a VLE triggering (blue) and failing to trigger (green) an action potential. Scale bar: 10 mV, 30 ms. The spike amplitude is truncated. ***C3***, The VLE maximum rise slope in the 30 consecutive responses. ***D1–D3***, ***E1–E3***, ***F1–F3***, Data show similar experiments for three other PC to FSBC (FSBC 3–5) pairs. Insets, Scaling as above, action potential amplitudes are truncated. Note that in the FSBC 3 and FSBC 5 the large VLEs at relatively negative postsynaptic Vm partially mask spike afterhyperpolarization.

Recordings were performed in a submerged chamber (perfused 8 ml/min) at 36–37°C. Cells were patched using water-immersion 20× objective with additional zoom (up to 4×) and infrared differential interference contrast video microscopy. In line with previous studies, VLEs were found in 10–15% of PC to interneuron connections tested (Molnár et al., 2008; Szegedi et al., 2016). Spike transmission data were obtained 10–30 min after break-in to whole cell. Micropipettes (5–8 MΩ) for whole-cell patch-clamp recording were filled with intracellular solution: 126 mM K-gluconate, 4 mM KCl, 4 mM ATP-Mg, 0.3 mM Na_2_–GTP, 10 mM HEPES, and 10 mM phosphocreatine (pH 7.20; 300 mOsm) with 0.3% (w/v) biocytin. Current- and voltage-clamp recordings were performed with a Mutliclamp 2B amplifier (Molecular Devices) or EPC 10 quadro amplifier (HEKA), and low-pass filtered at 6–8 kHz (Bessel filter). Series resistance (Rs) and pipette capacitance were compensated in current clamp mode and pipette capacitance in the voltage clamp mode. Rs was monitored and recorded continuously during the experiments. Voltage clamp recordings were discarded if the Rs was higher than 25 MΩ or changed by >20%. Spikes were generated in the presynaptic cell with brief (2–3 ms) suprathreshold depolarizing pulses in voltage clamp or current clamp mode (delivered every 10 s). Liquid junction potential error was corrected in all membrane potential values. Postsynaptic FSBC membrane potential in the experiments shown in Figure 1 was moderately depolarized (3.4 ± 1.7 mV, FSBC 1–4) or hyperpolarized (−5.2 mV, FSBC 5) from the resting membrane potential (−63.8 ± 3.6 mV, *n* = 5) recorded immediately after break-in to whole cell, aiming to adjust VLE-evoked spiking probability between the half-maximum and maximum in the cells. Accordingly, the non-FSINs were depolarized 11.6 ± 7.3 mV from the resting membrane potential (−68.7 ± 2.2 mV; *n* = 3). Membrane time constant and cell input resistance were measured in current clamp using −20 pA, 600− to 800-ms steps delivered at resting membrane potential. Firing frequency accommodation was tested by applying 600- to 800-ms depolarizing current steps to evoke firing between 30 and 40 Hz during the first 100 ms of the step. The non-FSIN 3 fired only single action potentials in response to the depolarizing steps, tested up to −20 mV.

### Data analysis and statistics

**Figure 2.**
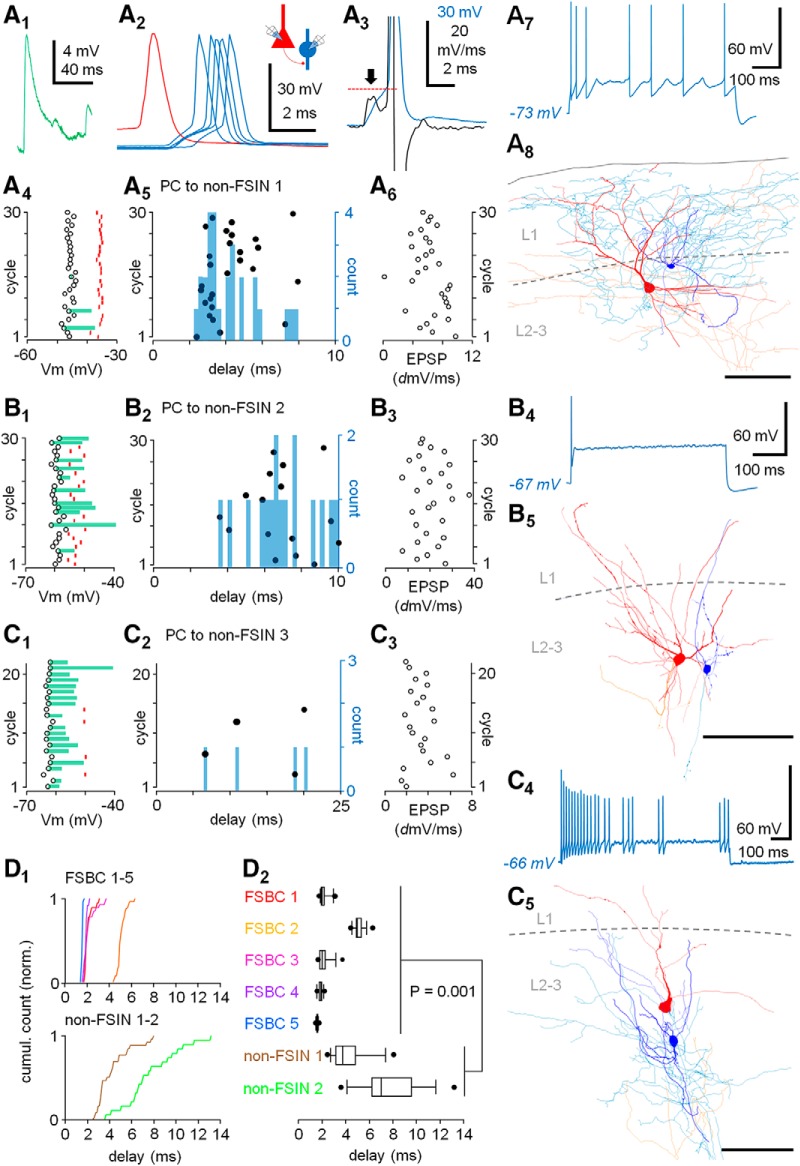
A PC spike triggers firing in non-FSINs with short delay but low temporal fidelity. ***A–C***, Three paired recordings showing PCs connected monosynaptically to non-FSINs, although VLEs that trigger their firing. ***A***, A PC connected to non-FSIN 1. ***A1***, Average of VLEs (three) that failed to fire. ***A2***, VLEs with action potential in five events (blue) triggered by solitary PC spikes (10-s interval, a sample shown in red). Schematic shows the experimental design. ***A3***, Derivative (black line) of a VLE with action potential (blue line). Arrow shows the VLE maximum rise slope, and the following hump in the black line marks the action potential onset. The onset membrane potential in the blue line is indicated by horizontal dotted red line. ***A4***, Firing evoked by the VLEs for 30 consecutive PC spikes (10-s interval). Open circles indicate the interneuron membrane potential (Vm), red marks show Vm for the triggered action potential onsets. Green bars show amplitude of the VLEs that failed to trigger firing. ***A5***, Timing of the non-FSIN 1 firing (black dots) in the 30 cycles. Blue histogram summarizes the evoked spike delay (count, bin 0.25 ms). ***A6***, The VLE maximum rise slope shows large trial-to-trial variability (30 consecutive cycles), including EPSP failure in cycle 15. ***A7***, The non-FSIN 1 firing response to a sustained depolarizing (600 ms) step shows clear firing frequency accommodation. Scale bar: 60 mV, 100 ms. ***A8***, Illustration of the presynaptic PC (soma and dendrites red, axon orange) and the postsynaptic non-FSIN 1 (blue, axon light blue). L1 and L2-3: layer 1 and 2–3, respectively. Scale bar: 100 μm. ***B***, Similar analyses of another PC to non-FSIN (non-FSIN 2) pair with VLEs. ***B1***, The non-FSIN 2 membrane potential (Vm, open circles) in 30 consecutive cycles of PC spikes (10-s interval). Red marks show Vm for onsets of the postsynaptic action potentials. Green bars illustrate amplitude of the VLEs that failed to fire. ***B2***, Timing of the firing (black dots) in the 30 cycles. Histogram (bin 0.25 ms) summarizes the spike delay distribution. ***B3***, The VLE maximum rise slope in the consecutive cycles. Note large trial-to-trial variability. ***B4***, The non-FSIN 2 shows just single action potential for a (600 ms) depolarizing pulse. Scale bar: 60 mV, 100 ms. ***B5***, Illustration of the PC (soma and dendrites red, axon orange) and the postsynaptic non-FSIN 2 (blue, axon light blue). L1 and L2-3: layer 1 and 2–3, respectively. Scale bar: 100 μm. ***C***, Analyses of a PC to non-FSIN 3 pair with VLEs. ***C1***, The Vm (open circles), the membrane potential for the action potential onset (red marks), and the amplitude of VLEs that failed to fire (green bars) in consecutive (22) cycles. ***C2***, Timing of the firing (black dots) and a histogram (bin 0.25 ms) summarizing the postsynaptic spike delay. ***C3***, The VLE maximum rise slope for the cycles shows again notable trial-to-trial variability. ***C4***, The interneuron firing shows clear firing frequency accommodation to a sustained depolarizing pulse. Scale bar: 60 mV, 100 ms. ***C5***, Illustration of the cell pair. ***D***, The FSBCs show shorter average spike delay and smaller spike delay variance than the non-FSINs. ***D1***, Cumulative histograms showing the VLE-evoked spike delays in the FSBC 1–5 and in the non-FSIN 1 and the non-FSIN 2. Spike delay data from the non-FSIN 3 was omitted here, because the experiment showed only four data points and most of them with longer than 10-ms delay. ***D2***, The spike delay values in the cells showing each individual neuron delay median, interquartile range, 5 and 95 percentiles, and the minimum and maximum values. Mann-Whitney test shows significant difference between the spike delay values of the FSBCs and of the non-FSIN 1–2. The non-FSIN 3 is omitted in the test because of the very low number of evoked spikes compared to the other cells.

Data were acquired with Clampex (Molecular Devices) or with PatchMaster software (HEKA) and digitized at 20–100 kHz. The data for EPSPs, IPSPs/Cs, action potential timing, axon current width, and the cell membrane time constant were analyzed off-line with p-Clamp (Molecular Devices, RRID: SCR_011323), Spike2 (version 8.1, Cambridge Electronic Design, RRID: SCR_000903), OriginPro (OriginLab, RRID: SCR_00281), IgorPro (WaveMetrics, RRID:SCR_000325), and SigmaPlot (RRID: SCR_003210) softwares. Data are presented as mean ± SEM, and for data with nonparametric distribution as median with lower and upper quartiles (interquartile range). For cell groups, the data are calculated from the means of individual experiments (mean of means). Monosynaptic IPSCs, and di- and multisynaptic IPSCs were filtered off-line using RC low-pass with cutoff frequency corresponding to 80-μs τ. The VLE average amplitude values were calculated for each cell from the VLEs that failed to spike in the experiments. In the experiments where spiking probability was high, additional VLEs were measured in a subthreshold potential to yield at least 3 VLEs for each cell to calculate the mean. For dV/dt analyses of monosynaptic VLEs, a 0.12-ms sliding average window was used to measure trace derivatives. The maximum VLE rising slope was measured from the derivative by averaging data points within 0.3 ms of positive peak. Postsynaptic action potential onset in VLE-spike complexes was identified using the response derivative, and membrane potential at this time point was used to define the firing threshold shown in Figures 1, 2. Evoked action potential delay to the presynaptic spike was calculated as a temporal distance of the pre- and postsynaptic spike peak. Spike kinetics in the interneurons were measured as the axon potential depolarizing phase width at half-maximal amplitude (in current clamp experiments), and as axon inward current width (in voltage clamp experiments).

VLE amplitude and time-to-peak, IPSPs and monosynaptic IPSCs were analyzed as described previously (Szegedi et al., 2016). For the IPSCs, derivative analysis was used to help to define the IPSC onset and the peak of individual IPSCs in the complex events (Fig. 4*B2*,*B3*). Rather than measuring the maximal rise slope directly from the IPSC derivative (because of small signal amplitude compared the noise in the slow IPSCs), the IPSCs were confirmed by visual inspection and fitted with slope curve for rise slope (20–80%) analysis. The IPSC rise slope was divided by the IPSC amplitude to define the amplitude-normalized rise kinetics. In the experiments measuring the monosynaptic IPSCs (amplitude-) normalized rise slope values, the rise slope value inversely correlated with the IPSC amplitude, suggesting that the variation observed in the normalized slope values in individual cells possibly emerged from asynchrony of released vesicle quanta (*r* = −0.73, *p* = 0.0000002, *n* = 60 IPSCs in six FSBCs, Spearman’s rank order correlation; Mody et al., 1994). For statistical analysis, ANOVA on ranks with Dunn’s multiple paired comparison (*post hoc* test), Mann--Whitney *U* test (MW test), and *t* test were used. Differences were accepted as significant if *p* < 0.05. Parametric distribution was tested with Shapiro-Wilk test using SigmaPlot (RRID: SCR_003210).

### Cell visualization and reconstruction

After electrophysiological recording, slices were immediately fixed in a fixative containing 4% paraformaldehyde and 15% picric acid in 0.1 M phosphate buffer (PB; pH 7.4) at 4°C for at least 12 h, then stored at 4°C in 0.1 M PB with 0.05% sodium azide as a preservative. Slices were embedded in 10% gelatin and further sectioned into slices of 50-μm thickness in the cold PB using a vibratome VT1000S (Leica Microsystems). After sectioning, the slices were rinsed in 0.1 M PB (3 × 10 min) and cryoprotected in 10–20% sucrose solution in 0.1 M PB. Finally, they were incubated in fluorophore (Cy3)-conjugated streptavidin (1:400, Jackson ImmunoResearch) in 0.1 M Tris-buffered saline (TBS; pH 7.4) for 2.5 h (at 22–24°C). After washing with 0.1 M PB (3 × 10 min), the sections were covered in Vectashield mounting medium (Vector Laboratories), put under cover slips, and examined under an epifluorescence microscope (Leica DM 5000 B). Sections selected for immunohistochemistry and cell reconstruction were dismounted and processed as explained below in Immunohistochemistry. Some sections for cell structure illustrations were further incubated in a solution of conjugated avidin-biotin horseradish peroxidase (ABC; 1:300; Vector Laboratories) in TBS (pH 7.4) at 4°C overnight. The enzyme reaction was revealed by the glucose oxidase-DAB-nickel method using 3’3-diaminobenzidine tetrahydrochloride (0.05%) as chromogen and 0.01% H_2_O_2_ as oxidant. Sections were further treated with 1% OsO_4_ in 0.1M PB. After several washes in distilled water, sections were stained in 1% uranyl acetate and dehydrated in ascending series of ethanol concentration. Sections were infiltrated with epoxy resin (Durcupan) overnight and embedded on glass slides. For the cells visualized in the figures, three-dimensional light microscopic reconstructions from one or two sections were conducted using the Neurolucida system (RRID:SCR_001775) with 100× objective (Olympus BX51, Olympus UPlanFI). Images were collapsed in the *z*-axis for illustration. FSINs in Figure 4*A2* referred to as unidentified FSINs (uFS) were unsuccessfully recovered for anatomic analysis.

### Immunohistochemistry

Free-floating sections were washed three times in tris-buffered saline containing 0.3% Triton X-100 (TBS-TX 0.3%) (15 min) at 22–24°C, then moved in 20% blocking solution with horse serum in TBS-TX, 0.3% for parvalbumin (pv) staining and 10% blocking solution for vesicular GABA transporter (vgat) staining. The sections were incubated in primary antibodies diluted in 1% serum in TBS-TX 0.3% over three nights at 4° C, then put in relevant fluorochrome-conjugated secondary antibodies in 1% of blocking serum in TBS-TX 0.3% overnight at 4° C. Sections were washed at first step in TBS-TX 0.3% (3 × 20 min) and later in 0.1 M PB (3 × 20 min) and mounted on glass slides with Vectashield mounting medium (Vector Laboratories). The characterizations of antibodies: pv (goat anti-pv, 1:500, Swant, AB_10000343) and vgat (rabbit anti-vgat, 1:500, Synaptic Systems, AB_887871). Fluorophore-labeled secondary antibodies were: DyLight 488 (donkey anti-goat, 1:400, Jackson ImmunoResearch), Alexa Fluor 488 (donkey anti-rat, 1:400, Jackson ImmunoResearch), and Cy5 (donkey anti-rabbit, 1:500, Jackson ImmunoResearch). Labeling of neurons by biocytin and immunoreactions were evaluated using first epifluorescence (Leica DM 5000 B) and then laser scanning confocal microscopy (Zeiss LSM880). The micrographs presented are confocal fluorescence images.

## Results

### Single PC spikes trigger FSBC discharges with short delay and high temporal precision through VLEs

First, we studied firing of FSBCs evoked by single spikes in layer 2–3 master PCs. We recorded synaptically connected PC to FSBC pairs in whole-cell clamp to find master PCs generating VLEs (average amplitude 13.4 ± 3.0 mV, *n* = 5 cell pairs, mean of means) and to study spike transmission in this specific neuronal circuit (Fig. 1*A1*). Solitary presynaptic PC spikes (interval 10 s) triggered single action potentials in the postsynaptic FSBCs, and the FSBC firing was abolished by postsynaptic hyperpolarization in all cell pairs studied (to −73.3 ± 5.2 mV, *n* = 5 cell pairs, mean of means) indicating that the spikes were triggered by the VLEs (Fig. 1*A2*). The postsynaptic interneurons exhibited narrow spike width (half-width 0.32 ± 0.05 ms, *n* = 5 cells, mean of means) and little firing frequency accommodation during a suprathreshold depolarizing step (Fig. 1*A3*; Szegedi et al., 2016; Wang et al., 2016). The interneurons were filled with biocytin and they showed axon forming boutons around L2-3 cell somata (Fig. 1*A3*,*A4*; Molnár et al., 2008; Blazquez-Llorca et al., 2010). One cell was tested for pv and found to be immunopositive (Fig. 1*A4*). The FSBCs were recorded in tissue material resected from frontal or temporal lobe as reported in detail in Table 1.

The master PC spike (interval 10 s)-evoked action potential in the FSBCs (Vm = −61.2 ± 3.2 mV, *n* = 5 cells, mean of means) showed short (2.67-ms average) delay (*n* = 117 spikes in 150 cycles of 5 cell pairs, 30 cycles in each) relative to the PC spike with 0.78 ± 0.10 probability (Fig. 1*B–F*). The evoked firing in the five FSBCs (FSBC 1–5), the VLE amplitudes that failed to trigger the spike, and the firing threshold for 30 consecutive PC spike cycles are shown in Figure 1*B1*–*B4* (FSBC 1) and Figure 1 *C1–F1* (FSBC 2–5). The FSBC firing delay results are depicted in raster plots and summarized with histograms in Figure 1*B5* (FSBC 1) and Figure 1*C2–F2* (FSBC 2–5).

The VLEs were stabile over the consecutive cycles of PC spikes (30 cycles) and showed 1.56 ± 0.27 ms time-to-peak (*n* = 5 cells, mean of means) and the maximum rise slope of 32.46 ± 1.12 mV/ms (*n* = 150 events in 5 cells) with small trial-to-trial variation of the slope (cv slope = 0.15 ± 0.02, *n* = 5 cells). The VLE rise-slope values and their stability for the 30 consecutive cycles in the FSBCs are illustrated in Figure 1*B6* (FSBC 1) and Figure 1*C3–F3* (FSBC 2–5).

To investigate whether the master PC-evoked firing varies between different type of interneurons, we recorded from three cell pairs where a master PC elicited firing in a non-FSIN through VLEs (Fig. 2*A1–A3*; Table 1). Unlike the FSBCs, these interneurons had slow spike kinetics (spike half-width 0.51± 0.06 ms, *n* = 3 cells, mean of means; DeFelipe et al., 2013; Szegedi et al., 2016). The VLEs (interval 10 s) evoked maximally single action potential (Vm = −56.2 ± 5.4 mV, *n* = 3, mean of means) with 6.35-ms average delay to the PC spike (43 spikes in 82 cycles, in three cells) at 0.58 ± 0.22 probability (*n* = 3). Panels Fig. 2*A4* (non-FSIN 1). The results are illustrated in Figure 2 as follows: Figure 2*B1* (non-FSIN 2) and Figure 1*C1* (non-FSIN 3) illustrate the VLE-evoked firing, the amplitude of VLEs failing to trigger the spike, and the firing threshold for the three non-FSINs in the consecutive cycles (30 cycles in non-FSIN 1 and 2, 22 cycles in non-FSIN 3). The VLE-evoked firing delay is illustrated in raster plots and summarized with histograms in Figure 2*A5* (non-FSIN 1) and Figure 2*B2*,*C2* (non-FSIN 2 and 3, respectively). Although the average VLE amplitude in the non-FSINs (9.7 ± 0.9 mV, *n* = 3 cells, mean of means) was not different from the VLEs observed in the FSBCs (*p* = 0.39, MW-test), the VLEs in non-FSINs had slower time-to-peak (5.78 ± 0.61 ms, *n* = 3 cells, mean of means, *p* = 0.038, MW-test) and lower maximum rise slope (9.28 ± 1.01 mV/ms, *n* = 82 events) than in the FSBCs (*p* = 0.001, *n* = 150 and 82 events, respectively, MW-test). In addition, the VLE rise slope trial-to-trial variation was larger in these cells (cv slope = 0.33 ± 0.06, *n* = 3) than in the FSBCs (*p* = 0.036, MW-test). The VLE rise slope values for the consecutive cycles are depicted for the non-FSINs in Figure 2*A6* (non-FSIN 1) and Figure 2*B3*,*C3* (non-FSIN 2 and 3, respectively). Each non-FSIN showed prominent firing frequency accommodation or generated just single spike to a sustained depolarizing step as illustrated in Figure 2*A7* (non-FSIN 1) and Figure 2*B4*,*C4* (non-FSIN 2 and 3, respectively). The cells were filled with biocytin and visualized, and they showed multipolar somatodendritic structure with dendrites lacking spines. Reconstructions of the three PC to non-FSIN pairs are illustrated in Figure 2*A8* (non-FSIN 1) and Figure 2*B5*,*C5* (non-FSIN 2 and 3).

Next, we compared the VLE-evoked spike delay between the FSBCs and the non-FSINs. In the FSBCs, the median spike delay varied between the cells from 1.61 ms (FSBC 5) to 5.0 ms (FSBC 2), and in the non-FSINs from 3.73 ms (non-FSIN 1) to 14.7 ms (non-FSIN 3). Altogether, the FSBCs showed shorter spike delay (median and interquartile range: FSBCs = 1.96, 1.68–3.25, non-FSINs = 5.60, 3.35–7.70, *n* = 117 and 43 spikes, respectively) and smaller spike delay variance than the non-FSINs (*p* = 0.001, MW-test). The FSBCs had membrane time constant of 8.6 ± 0.8 ms (*n* = 5) and the non-FSINs 7.2 ± 4.2 ms (*n* = 3). The spike delay values in the two cell groups are shown in detail in Figure 2*D1* with individual neurons’ delay median, interquartile range, 5 and 95 percentiles, and the minimum and the maximum values (Fig. 2*D2*), and statistical comparison of all spike delay values between the FSBCs and the non-FSIN 1–2. (The non-FSIN 3 was omitted in the analysis because of clearly lower number of spikes in the experiment compared to others.)

Thus, in the human neocortex layer 2–3 FSBCs show high fidelity “fast in-fast out” spike transmission (Hu et al., 2014) triggered by solitary master PC spikes. In addition, the master PC-triggered firing precision varies between layer 2–3 interneurons types, and the high precision discharge of the FSBCs is not seen in all interneuron types.

### GABAergic interneuron discharge in the complex events is time locked to master PC spike with a short interneuron-specific delay

**Figure 3.**
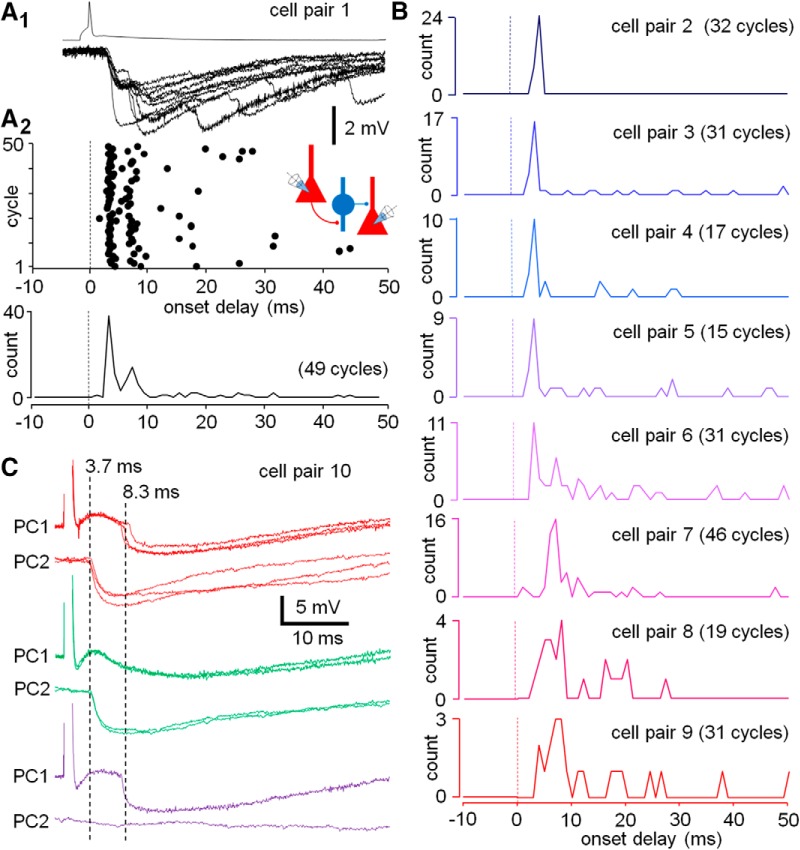
A PC spike triggers GABAergic synaptic events with short delay and high temporal precision**. *A***, Simultaneous recording from two PCs demonstrates that solitary PC spikes elicit time-locked GABAergic IPSPs with a few millisecond onset delay. A sample recording in the cell pair 1 shows single PC spike-evoked GABAergic IPSPs with two predominant delays during first 10 ms of the triggered activity. The IPSPs with the distinct delays occur successively in individual complex event episodes. ***A1***, A PC spike and 10 consecutive complex event episodes showing IPSPs (at –55 mV). ***A2***, Plot shows timing of the IPSPs. Dots indicate the IPSP onset delay to the PC spike in consecutive cycles (49 cycles, PC spike interval 10 s). Schematic shows the experimental design. Histogram summarizes the IPSP count against the IPSP onset delay (bin 1 ms). ***B***, Line histograms show IPSP onset delays in eight similar PC-PC pair recordings (cell pairs 2–9) as shown in ***A***, illustrated here in different colors. Ordinates show the IPSP count. From top down, the experiments first show patterns with occurrence of single delay peak (cell pairs 2–5), then complex pattern activity where the short-delay peak is followed by IPSPs with longer delay and lower probability (cell pairs 6–7), and finally cell pairs (8–9), where the complex events are comprised of loosely time-locked IPSPs occurring at low probability. ***C***, IPSPs are time locked to PC spike with pathway-specific delays. Recording from a PC-PC pair (cell pair 10) shows two IPSPs time locked to PC1 spike (interval 10 s) with average delay of 3.7 ms (in PC2) and 8.3 ms (in PC1). The IPSPs are generated by separate interneurons as revealed by cycles showing independent failures in either PC1 (green) or in PC2 (magenta).

Next, we examined discharge of GABAergic interneurons in complex events while avoiding direct microelectrode recording from the cells (Komlosi et al., 2012), since it can potentially alter their excitability and the firing response to VLEs. We measured master PC spike-evoked IPSPs in L2-3 PCs. We analyzed the onset delay to the PC spike of 357 IPSPs (in 50-ms time window after a PC spike evoked every 10 s) recorded in 9 PC-PC pairs (269 cycles, 15–49 cycles per pair) most of them in the frontal or temporal cortex (Table 1). The occurrence of IPSPs during the complex events in the experiments is summarized in Figure 3*A*,*B*. The majority of the IPSPs (*n* = 281 IPSPs) occurred during the first 10 ms of the complex events. These IPSPs were generated with high probability (0.87 ± 0.03 for the occurrence of IPSP in first 10 ms, *n* = 281 cycles in 9 cell pairs). In six experiments, the predominant IPSP onset delay was <5 ms (3.8 ± 0.2 ms, in 185 cycles in six pairs). In three experiments, the main delay was longer and between 5 and 10 ms (7.9 ± 0.3 ms, *n* = 65 IPSPs in 96 cycles in three pairs, MW-test; IPSP probability in first 10 ms 0.85 ± 0.02; Fig. 3*B*). In addition, various complex event episodes exhibited two or more IPSPs with distinct delays, with the later IPSPs showing lower probability than the first one (Fig. 3*A,B*). Because in the experiments in Figure 1 we had found that the master PCs elicited only single action potential in the GABAergic cells, we hypothesized that the occurrence of two or more IPSPs in same complex event episodes might emerge from separate GABAergic cells. Accordingly, we performed an experiment recording master PC-evoked IPSPs in two postsynaptic PCs simultaneously, but showing statistically different delays (3.72 ± 0.27 and 8.34 ± 0.23 ms, *n* = 8 and 6 IPSPs evoked in 14 cycles; *p* = 0.002, *t* test) and failures independent of each other (Fig. 3*C*). The results indicate that the IPSPs emerged from the firing of distinct individual interneurons. Altogether, the results on the IPSPs in the PC-PC pairs demonstrate that master PC spikes trigger high fidelity discharge of some GABAergic interneurons with short and specific delay.

### GABAergic synaptic currents with distinct kinetics manifest the activation of different interneuron subpopulations in complex events

**Figure 4.**
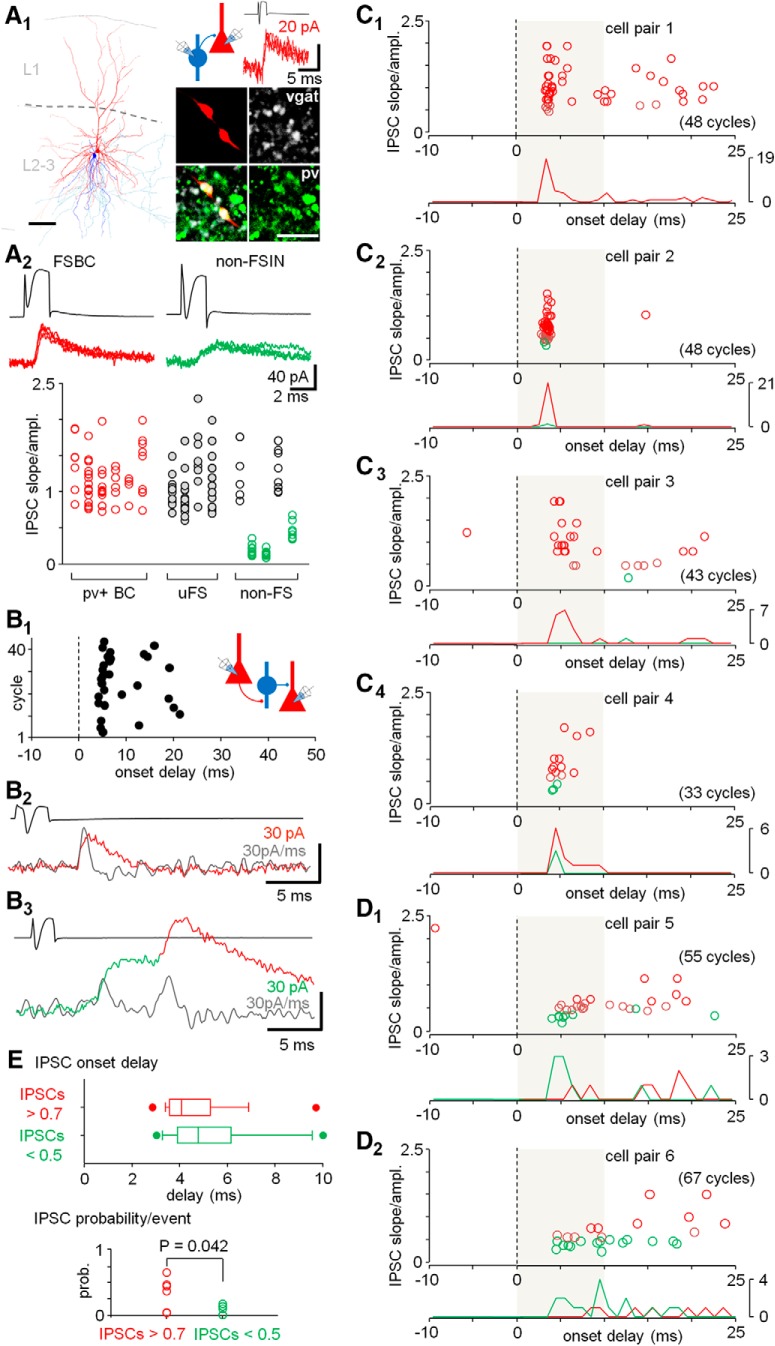
GABAergic synaptic currents with fast or slow rise slope reveal the discharge of different interneurons in the complex events. ***A***, GABAergic synaptic currents from FSBCs and some non-FSINs show different kinetic features in L2-3 PCs. ***A1***, One visualized synaptically connected FSBC (blue, axon light blue) to PC pair (red, axon orange). L1 and L2-3: layers 1 and 2–3, respectively. Scale bar: 100 μm. Insets, Schematic summarizes the experimental design. Traces show four superimposed consecutive monosynaptic IPSCs in the postsynaptic PC (red traces, at –55 mV) evoked by the FSBC spikes (black trace, interval 10 s). Micrographs illustrate pv+ (Alexa Fluor 488) and vgat+ (Cy5) axon boutons of the biocytin-filled (Cy3) presynaptic FSBC. Scale bar: 5 μm. ***A2***, Monosynaptic IPSCs evoked from FSBCs and some non-FSINs to PCs show distinct IPSC rise slope kinetics. Top, Sample monosynaptic IPSCs (four) in postsynaptic PCs (red traces, at –55 mV) evoked by spikes (single traces shown in black) of a FSBC or a non-FSIN. Bottom, Plot shows monosynaptic IPSC rise slope kinetics (IPSC rise slope normalized by the amplitude) in 15 interneuron to PC pairs. The value variation in individual cells correlates inversely with the IPSC amplitude indicating it emerges from release asynchrony (see Materials and Methods). Red dots show IPSCs from identified FSBCs. Green dots show slow IPSCs exclusively evoked from non-FSINs. pv+ BC, pv immunopositive FSBCs; uFS, fast-spiking cells not successfully visualized and identified; non-FS, nonfast spiking cells. ***B–D***, Recordings from PC-PC pairs show network-driven IPSCs with distinct rise slope kinetics in the complex events. ***B1***, A sample recording in voltage clamp (at –55 mV) showing the occurrence and the delay of a PC spike (10-s interval, 43 cycles)-evoked IPSCs (same experiment as the cell pair 3 below). ***B2***, Sample trace in one experiment showing a fast network-driven IPSC (red) defined by the high-rise kinetics. The IPSC derivative is shown in gray. ***B3***, Sample trace showing an evoked slow kinetic IPSC (green) followed by a fast IPSC (red) in a complex event. ***C1–C4***, PC spike-evoked complex events showing predominantly IPSCs akin to those generated by the FSBCs with fast rise-slope (red, rise slope to amplitude ratio >0.7) in the beginning (during first 10 ms) of the events. Green dots indicate IPSCs with slow rise slope akin to those generated exclusively by the non-FSINs (ratio <0.5). IPSCs with the amplitude-normalized rise slope value from 0.5–0.7 are indicated in brown. The plots show the IPSC amplitude-normalized rise slope value (ordinate) versus the IPSC delay (abscissa, 0 time point indicates timing of the master PC spike). The line histograms (bin 1 ms) below summarize the delay distribution of the fast (red, ratio >0.7) and the slow (green, ratio <0.5) IPSCs in each experiment (number of the cycles shown in parentheses). Line histogram ordinate shows count. The early complex event (first 10 ms) in the plots is marked with shaded background. ***D1***, ***D2***, Similar dot plots and histograms from two PC-PC pair recordings showing complex events with predominantly slow IPSCs (ratio <0.5) and only occasional fast IPSCs. ***E***, top, Summary of the onset delay values of the fast IPSCs (ratio >0.7, red) and the slow IPSCs (ratio <0.5, green) pooled in all 294 complex events in the six experiments in early phase of the complex events (during first 10 ms). Box plot shows median, interquartile range, 5 and 95 percentiles and the minimum and maximum measured in the first 10 ms of the events. Bottom, Plot shows higher probability of the fast IPSCs (events/cycle) than the slow IPSCs in the six experiments (*t* test). Individual dots show the probability in the individual experiments.

Finally, we studied if the GABAergic synaptic activity during the master PC-evoked complex events would reflect the VLE-evoked discharge of the FSBCs as demonstrated in Figure 1. As above, the experiments were performed in tissue samples mostly from the frontal or the temporal cortices (Table 1). First, to investigate kinetic properties of distinct GABAergic neuron type-evoked IPSCs, we recorded from 15 monosynaptic interneuron to PC pairs in voltage clamp (at – 55 mV). In the connections from FSINs (inward axon current width 0.45 ± 0.02 ms) to PCs, the IPSCs were 27.6 ± 2.2 pA in amplitude with 33.0 ± 1.9 pA/ms rise slope (*n* = 10 cell pairs, mean of means). Six of the successfully visualized FSINs were tested for pv and the vgat and found to be immunopositive for both (Fig. 4*A1*). The six FSINs were identified as basket cells. In turn, monosynaptic IPSCs (average amplitude 22.0 ± 3.3 pA, *n* = 5) from non-FSINs (inward axon current width 0.93 ± 0.06 ms, *n* = 5 cells, mean of means, *p* = 0.003 compared to the FSINs, *t* test) to PCs showed wide range of IPSC rise slope values in the studied pairs. In two non-FSIN connections to PC, the IPSCs were indistinguishable from those evoked by the FSINs (Fig. 4*A2*), and in three connections the IPSCs showed distinctly slower rise slope (6.2 ± 3.8 pA/ms, *n* = 3, mean of means) than generated by any of the FSINs (*p* < 0.05 for each non-FSIN, ANOVA on ranks, Dunn’s pairwise *post hoc* test with at least five events in each tested pair). The slope values in each recording were normalized by the IPSC amplitude to exclude any variation in the rise slopes possibly emerging from small differences in the IPSC electrochemical driving force between individual experiments. The normalized rise slope of the FSIN-evoked currents was 1.25 ± 0.11 (*n* = 10 pairs, mean of means). The IPSCs from the non-FSINs had significantly slower normalized rise slope of 0.27 ± 0.10 (*n* = 3 pairs, mean of means; *p* < 0.05 for each non-FSIN in ANOVA on ranks and Dunn’s pairwise *post hoc* test against the FSINs with at least five events in each tested pair). The amplitude-normalized IPSC slope values for all cells are shown in Figure 4*A2*.

As the amplitude-normalized IPSC rise slope provides a robust tool to discriminate the fast IPSCs generated by FSBCs (and some non-FSINs) and the slow IPSCs emerging exclusively from non-FSINs, we investigated the IPSC rise slope in network activity episodes evoked by master PC single spikes (10-s interval; Fig. 4*B1*–*B3*). The IPSCs in complex events had 24.4 ± 2.4 pA average amplitude (mean of means in six experiments), akin to the monosynaptic IPSCs in the 15 cell pairs studied above (*p* = 0.91, MW-test). We categorized complex event IPSCs by the amplitude-normalized rise slope value as shown by the monosynaptic IPSCs: the ratio >0.7 similar to the IPSCs monosynaptically evoked by FSBCs as illustrated red in Figure 4*A2*, the ratio <0.5 corresponding to IPSCs exclusively evoked by non-FSINs (Fig. 4*A2*, green), and ratio 0.5–0.7 falling in between the two as defined in Figure 4*A2*. The occurrence of IPSCs and their rise slope (normalized by the amplitude) were analyzed in the complex events of six experiments as illustrated with sample traces in Figure 4*B1*–*B3*.

We found that in four experiments the network-driven IPSCs with mainly fast amplitude-normalized rise slope (1.05 ± 0.10 average of all IPSCs in first 10 ms of complex events, *n* = 117 IPSCs in 172 complex events in four cells) predominated activity (Fig. 4*C1*–*C4*). When we focused the analysis on the first 10 ms (corresponding to monosynaptic spike time window observed in the FSBCs earlier), the fast rise time (>0.7) IPSCs occurred in 84 cycles of the 172 cycles and showed 3.94-ms delay (median with 3.55- to 5.20-ms interquartile range). The slow kinetic IPSCs (<0.5) occurred only in two experiments (Fig. 4*C2*,*C4*) with low probability (nine events in 172 cycles) in the same time window.

However, we found that in two experiments mostly slow kinetic IPSCs were generated, although with low probability in the first 10-ms time window (14 in 122 cycles) and only very few fast kinetic IPSCs events (four in 122 cycles) occurred in the early (first 10 ms) of the events (Fig. 4*D1*,*D2*).

In order to compare the temporal distribution and the probability of the two types of IPSCs (the fast and the slow) in the early complex events, we pooled the IPSCs in all 294 cycles of the six experiments. The results are illustrated in Figure 4*E* showing no difference between the delay (*p* = 0.095, MW-test) of the fast IPSCs (median and interquartile range: 4.08 and 3.56–5.30 ms, respectively) and the slow IPSCs (4.78 and 4.01–6.10 ms), but demonstrating higher probability (*p* = 0.042, *t* test) of occurrence of the fast than the slow IPSCs in the early (first 10 ms) complex events.

In conclusion, the results demonstrate that IPSCs akin to those generated by FSBCs regularly occur with a short delay and high temporal fidelity in the beginning of the complex events in the experimental conditions that avoid direct recording from interneurons. In addition, the experiments show that discharge of many non-FSINs occurs at low probability.

## Discussion

Strong VLE-synapses from some layer 2–3 PCs to GABAergic interneurons represent a distinctive microcircuit feature in the human neocortex allowing these master PCs to initiate tens-of-millisecond-long discharge in the local neuronal network by single action potentials (Molnár et al., 2008; Brecht, 2012; Lourenço and Bacci, 2017). Here, we show that fast-spiking GABAergic basket cells are regularly activated at the beginning of these events with short delay and high temporal precision.

Microcircuits generating the complex events apparently represent a common feature in the human neocortex. The VLEs occur in ∼10–15% of the PC to FSIN synapses and the single PC spike-evoked interneuron firing has been reported in various neocortical areas in tissue samples resected from human subjects varying in age and gender (Molnár et al., 2008; Komlosi et al., 2012; Molnar et al., 2016). Although the specific function of the complex events, as characterized in the brain slices, is still unknown, the VLE-evoked accurate discharge of the FSBCs and the disynaptic inhibition transferred from these interneurons could contribute to generation of coordinated network oscillations where FSBCs play a key role (Cunningham et al., 2004; Hájos et al., 2004; Ellender and Paulsen, 2010; Florez et al., 2015; Averkin et al., 2016). Importantly, unlike rodents the human neocortical microcircuits can trigger basket cell firing by a single master PC action potential, and this may provide an important computational feature in cortical processing in the human compared to rodents (Lourenço and Bacci, 2017).

The temporal fidelity of the synaptically-triggered basket cell firing and the fast kinetic time-locked IPSCs in the early complex events reflect basket cells fast-in-fast-out signaling feature akin to characterized in these interneurons in rodents (Hu et al., 2014). The VLEs in the basket cells showed short time-to-peak value and a remarkably fast rise slope, which together with their short membrane time constant can explain the short delay of the synaptically evoked spikes. The VLE synapses to FSINs have high release probability (Molnar et al., 2016), and this feature is in line with the observation here that the VLE rise slope value showed little variation in consecutive cycles in the FSBCs. This further increases temporal precision of the VLE-evoked spikes and explains their small jitter. In addition, the remarkably narrow time window of the VLE-evoked basket cell firing and the observation that only single spikes were generated by each VLE, may be set by autaptic GABAergic inhibitory synapses or GABAergic connections from other interneurons to these cells (Tamás et al., 1997; Hioki et al., 2013; Deleuze et al., 2014; Lourenço et al., 2014). Curiously, although one FSBC (FSBC 2) showed slightly longer average spike delay than the other basket cells investigated here, it along with the others also showed small spike delay jitter. The master PC-evoked firing of the non-FSINs showed lower temporal fidelity than the basket cells. This can be partly explained by the large trial-to-trial variation of the VLEs and the long VLE time-to-peak in the non-FSINs. Postsynaptic membrane potential and the VLE amplitude also regulate spike transmission (Kretzberg et al., 2001). Therefore, it is likely that these interneurons' input-output transformation is further controlled by brain state-dependent membrane potential fluctuations (Puig et al., 2008; Fanselow and Connors, 2010) and by plasticity of the VLEs (Szegedi et al., 2016).

Although this study almost entirely focuses on the FSBCs, it also shows that in addition other cortical interneuron types discharge in complex events. In particular, we demonstrated the discharge of non-FSINs with variable delay and low probability (Szegedi et al., 2016). However, the non-FSINs in general comprise a highly diverse group of interneuron types and a separate study will be needed in the future to investigate the firing behavior of identified non-FSIN cell types (Tremblay et al., 2016). In addition, fast-spiking axo-axonic cells fire with a short delay akin to the FSBCs reported here and these GABAergic cells can excite PCs and may trigger their firing though depolarising GABAergic effect on the axon initial segment (Szabadics et al., 2006; Molnár et al., 2008; Komlosi et al., 2012). In line with this, polysynaptic EPSCs are often generated in complex events with 5- to 10-ms delay to a master PC spike, apparently evoked by these interneurons (Molnár et al., 2008; Komlosi et al., 2012) since VLE-like synaptic contacts have not been found between L2-3 PCs (Molnár et al., 2008; Szegedi et al., 2016).

In a slice preparation, the complex event activity patterns can be deformed with partially pruned synaptic networks. This might explain why the fast- and the slow-kinetic IPSC occurrences showed very different patterns between some individual experiments here. Another more exciting possibility for this observation is that the distinct complex event structures genuinely reflect diversity of neuronal ensembles established in the brain before the resection of the cortical tissue. Although the hypothesis is challenging to address experimentally in humans, further investigation of distinct interneuron types discharge during the complex events will help to judge this idea.

To conclude, the results hitherto show that human cortical microcircuits generating complex events involve various specialized GABAergic interneuron types. We suggest that various cell types may show specific firing behavior during the events as we report here for the FSBCs (Klausberger and Somogyi, 2008). Therefore, the activation of FSBCs in early phase of the complex events may just represent one common feature in these human neocortex network activity episodes.

## Synthesis

Reviewing Editor: Pablo Castillo, Albert Einstein College of Medicine Decisions are customarily a result of the Reviewing Editor and the peer reviewers coming together and discussing their recommendations until a consensus is reached. When revisions are invited, a fact-based synthesis statement explaining their decision and outlining what is needed to prepare a revision will be listed below. The following reviewer(s) agreed to reveal their identity: Alberto Bacci, Arianna Maffei

Your manuscript was evaluated by two expert in the field. Here, I summarize the online discussion. Both reviewers agree on the high quality of the results and the significance of your findings as they provide important insights into the organization of local circuitry in human cortex. However, they think there are a number of points that should be clarified -no need to perform new experiments. They also indicate your manuscript is hard to read and needs to be edited, if possible, by a native English writer and all changes in the text should be properly marked. Below, please find the reviewers' specific comments.

REVIEWER#1:

In this manuscript, Szegedi and colleagues describe a specific circuit activity of the human neocortex, induced by single action potentials from specific layer 2/3 pyramidal neurons. These neurons are defined by the authors as "master PCs". The authors analyze unitary connections between pyramidal cells and FS and some non-FS interneurons. They found that FSBCs are key generators of fast and reliable di-synaptic events in downstream pyramidal neurons, due to strong and fast connectivity inducing reliable spike transmission between PCs and FSBCs.

The paper reports interesting data, obtained in human resected tissue, and therefore it bears important implications towards understanding human-specific features of cortical microcircuits. However, the manuscript could be improved, as in the present form it suffers from some limitations:

1. The manuscript should be improved in writing and data presentation. The text is often garbled, and difficult to read. It contains many grammatical mistakes and some sentences are obscure (One example of the many throughout the text: Page 4, line 76: "A temporally pattered discharge of GABAergic inhibitory interneurons is a hallmark of the network activity encoding such neuronal ensembles as has been demonstrated in animal studies, and the strongest synaptic connections govern the population activity in these operations. (Isaacson and Scanziani, 2011; Brecht, 2012; Buzsaki and Watson, 2012). Therefore, we hypothesize that the master PC-evoked complex events might exhibit temporally organized activity of interneuron types involved in these processes.").

The aim of the study should be better emphasized in the introduction, and the rationale of each experiment should be better spelled out in each sub-section of the Results. The Discussion muses on co-ordinated gamma or high gamma, but it is unclear why, as the study does not focus on network oscillations. The Discussion should instead interpret the actual results (see below). Likewise, figures are cluttered. The authors might want to consider including repetitions of the same results in the 'Extended data' section.

2. The number of observations is low, as the experiments are inherently difficult. Indeed, these recordings, analyses and anatomical reconstructions from human tissue are laudable, but I am unsure about the power of statistical tests run on n = 2 or 3.

3. In the Methods section, it is indicated that master PCs amount to 10-15%. This is a relatively low number, and thus the relevance the phenomenon should be properly discussed. What is the relevance of these relatively rare VLEs within cortical circuits? What could it be due to: human-specific PC types? Is there any morphological peculiarity of PCs exhibiting VLEs? Are the majority of PCs (not behaving like master PCs) behaving like in rodents? Moreover, VLEs occur also at non-FS interneurons: what is the occurrence?

4. Regarding the slower and smaller IPSCs recorded from non-FS interneurons: could their smaller strength and temporal coupling be due to dendritic filtering, instead of reduced discharge probability, as stated by the authors?

5. It is not clear why the slope of IPSCs was normalized to their amplitudes. What does this index represent, exactly?

6. Regarding the delayed inhibitory events, which occur late (several tens of ms) with respect to the spike fired by the master PC: how do the authors exclude that this activity might be due to spontaneous neurotransmission, independent of the stimulus?

7. The experiments were done using 3 mM extracellular Ca: how physiological is it? To my knowledge, the Ca concentration in human CSF is ~1 mM. Is there a reason for using 3 mM? Could this relatively high concentration change the presynaptic release properties of cortical PCs?

Minor issue: The authors use the term phase-locked, but there is no periodic activity and therefore no actual phase. I would consider changing 'phase-locked' with AP-locked (or locked to the stimulus).

REVIEWER #2:

The study explores the role of inhibitory neurons in the generation of complex events triggered by layer 3/4 pyramidal neurons in human neocortex. The authors perform a set of challenging electrophysiological recordings from slices of human tissue and provide a thorough data analysis unveiling how the activation of inhibitory neurons by single pyramidal neurons can modulate the temporal organization of cortical activity.

In my opinion, the authors have thoroughly addressed the concerns of the previous reviewers.

I only have a minor point: The abstract is rather technical and may make it difficult for some readers to extract a take-home message. As the manuscript is likely of interest to a broad audience, it may be in the interest of the authors to make it more approachable to non-physiologists.
